# A bibliometric analysis of NOAA’s Office of Ocean Exploration and Research

**DOI:** 10.1007/s11192-012-0836-0

**Published:** 2012-09-05

**Authors:** Chris W. Belter

**Affiliations:** LAC Group, NOAA Central Library, Silver Spring, MD 20910 USA

**Keywords:** Citation analysis, Bibliometric mapping, Research funding, Research evaluation, Science policy

## Abstract

Bibliometric analysis techniques are increasingly being used to analyze and evaluate scientific research produced by institutions and grant funding agencies. This article uses bibliometric methods to analyze journal articles funded by NOAA’s Office of Ocean Exploration and Research (OER), an extramural grant-funding agency focused on the scientific exploration of the world’s oceans. OER-supported articles in this analysis were identified through grant reports, personal communication, and acknowledgement of OER support or grant numbers. The articles identified were analyzed to determine the number of publications and citations received per year, subject, and institution. The productivity and citation impact of institutions in the US receiving OER grant funding were mapped geographically. Word co-occurrence and bibliographic coupling networks were created and visualized to identify the research topics of OER-supported articles. Finally, article citation counts were evaluated by means of percentile ranks. This article demonstrates that bibliometric analysis can be useful for summarizing and evaluating the research performance of a grant funding agency.

## Introduction

The Office of Ocean Exploration and Research (OER) at the US National Oceanic and Atmospheric Administration (NOAA) is an extramural grant funding agency focused on the exploration of the world’s oceans. OER was created in 2007 with the merger of NOAA’s Office of Ocean Exploration (OE) and National Undersea Research Program (NURP). OE was created in 2001 to facilitate the implementation of the US National Ocean Exploration Program at the recommendation of the Presidential Panel on Ocean Exploration convened in 2000. OE/OER’s goals are to facilitate the scientific exploration of the world’s oceans, to turn ocean discoveries into new applications that benefit society, to increase the efficiency of underwater exploration through the advancement of underwater technology, and to engage and educate the public. NURP, a network of regional undersea research centers, was created in 1982 to support the scientific study of regional ocean zones and the development of underwater technologies.

During its 10-year anniversary in 2011, OE/OER began the process of summarizing its program achievements and evaluating the degree to which its research funding and scientific programs were achieving its mission goals. As a part of this process, OER commissioned the author to perform a bibliometric analysis on all of the scientific publications that had been produced with OE/OER support over its first 10 years. Prior to this analysis, OER had little knowledge of what scientific research had resulted from its funding, which institutions had produced that research, and what the significance of that research was. The purpose of this analysis was to begin to address this need by identifying the topics, sources, and citation impact of publications supported by OE/OER. Results of this analysis can then be used to assist in the evaluation of funding decisions made by OER in order to ensure that the research produced with OER funding matches the areas of priority outlined in OER’s mission.

Bibliometric methods have often been used to analyze publications supported by grant-funding agencies like OER. In the medical sciences, the US National Institutes of Health (Boyack and Jordan 2011; Druss and Marcus 2005; Lyubarova et al. 2009), the UK Multiple Sclerosis Society (Rangnekar 2005), the Health Research Council of New Zealand (Gunn et al. 1999), and the Spanish Society of Cardiology and Spanish Heart Foundation (Benavent et al. 2011) have all sponsored bibliometric evaluations of their funding programs. Other bibliometric analyses have been performed on grant funding agencies in the chemical sciences (Jain et al. 1998), biological sciences (Jokic 2000; Porter et al. 2012), forest sciences (Klenk et al. 2010), and science policy (Zoss and Borner 2012). The author has been unable to locate a bibliometric analysis of a funding institution in the geosciences or environmental sciences.

Bibliometric analysis has also been used to measure the citation impact of authors and publications that have received grant support versus those that have not. A number of studies (Armstrong et al. 1997; Campbell et al. 2010; Lichtman and Oakes 2001) have found that authors who were awarded research scholarships or other awards were more productive and more highly cited than non-funded authors. Findings on the correlation between grant funding and citation impact of articles are more mixed. Cronin and Shaw (1999) found no relationship between funding and citation count of articles in information science journals. Bourke and Butler (1999) found that citation impact of biological research in Australia was not determined as much by the mode of funding as by the position of the researcher. However, Lewison and colleagues found that articles by UK authors on gastroenterology (Lewison 1998) and arthritis (Lewison and Devey 1999) that acknowledged grant support had a significantly higher citation impact than those that did not. More recently, Zhao (2010) found that grant-funded research in library and information science had significantly higher citation counts than those that did not acknowledge funding. In addition, Hall et al. (2012) found that transdisciplinary teams were more productive and collaborative with grant funding than individual investigators.

Scientific research funded by government agencies like OER also has broader societal impacts. Narin et al. (1997) found that research at public institutions that was funded by government agencies such as NSF and NIH was heavily cited by patents, suggesting that grant funding of basic scientific research by government agencies has a significant impact on technology development. Huang et al. (2005, 2006) also found that NSF-funded patents in nanotechnology tend to have a higher impact than those funded by other groups. In addition, Salter and Martin (2001) found that publicly funded research has significant economic benefits to the country that funded the research, but cautioned that these benefits can take many forms and are therefore difficult to summarize.

The purpose of the present article is to present a summary-level bibliometric analysis of the scientific journal articles produced with OE and OER support from 2002 to April 2012 using data derived from Web of Science, Science Citation Index Expanded (WoS). Although OER has supported the production of numerous types of scientific publications, including book chapters, technical reports, and conference proceedings, these other types of publications were omitted from this analysis. Institution, country, citation, and citing article data are not available for non-journal articles in WoS, so, rather than changing the underlying data set depending on the analysis, these other publication types were omitted.

In addition, this analysis focuses solely on articles supported by OE. Articles sponsored by regional NURP centers, including those published subsequent to the OE/NURP merger, are not included in this analysis. Emphasis in this analysis will be placed on describing the nature of OER-supported articles in order to identify the authors and institutions responsible for creating these articles, the major research areas represented in these articles, and the citation impact of these articles in the identified research areas.

It is important to note that while this analysis focuses on the journal articles produced with OER support, the research areas and priorities of these publications do not necessarily reflect the research priorities or funding decisions of OER. Rather, these publications reflect the productivity of the individual scientists who have received OER support. Attempting to measure the degree to which these publications reflect the mission priorities and funding decisions of OER is beyond the scope of this analysis.

## Methodology

For the purposes of this study, an “OER-supported article” is defined as any article that has received financial, logistical, or other support from OER to gather data for or to perform all or part of the analysis described in the article; any article that utilizes specimens, data, imagery, etc. collected on an OER-supported expedition; or any article authored or co-authored by an employee of OER. This study employs a “full counting method” of assigning publications to OER; that is, any article that receives support from multiple agencies, including OER, is counted as an OER-supported article.

### Article identification

OER-supported articles were identified using two complementary methods. In the first method, author-identification, one or more authors of each article confirms through personal communication or through a formal grant report that the article was written with support from OER. Technically, each grant recipient is required to submit a formal activities report for each grant received in which the recipient identifies the publications that have resulted from that grant. In practice, this requirement is not always observed, but these reports did at least provide an initial list of publications.

In order to increase the number of publications found by the author-identification method, OER systematically emailed all recipients of OER grant funding requesting that they submit lists of publications associated with each grant. The response rate to these emails was approximately 40 %, although many of the scientists who responded submitted publications by their colleagues and co-grantees in addition to their own works.

Although the author-identification method is often the only method used to identify articles produced with grant support, there were a number of limitations to this method in this case. First, incomplete grant reporting and the low email response rate meant that OER received an incomplete set of publications that suffered from a self-selection bias. Second, the articles that were identified did not all acknowledge support from OER, with the result that verifying the validity of some of these articles was not possible. Finally, some authors did not distinguish between articles supported by OER and those supported by NURP, calling into question the accuracy of some of these identifications.

In order to partially correct for some of these limitations, additional OER-supported articles were identified by a second method: by searching databases and full-text article repositories for either acknowledgement of OER support or listing of OER as an author affiliation. The search string used in this method was:

“noaa ocean exploration” OR “noaa’s ocean exploration” OR “office of ocean exploration” OR “noaa oe” OR “noaa oer”.

This search was executed in Web of Science to search both the “Funding Agency” and the “Author Address” fields. The search string was then used to query the websites of numerous publishers including Elsevier, Springer, Wiley, the American Geophysical Union, InterResearch, Oxford, Cambridge, Nature, Science, PNAS, and so on to identify acknowledgement and funding text not available in WoS. The same search was also executed in Google Scholar. Each search result was manually verified in order to ensure that the word match indicated an acknowledgement of support by OER and not a citation to an OER publication or a discussion of OER activities.

A second round of searches was also performed to find OER grant numbers. Since some articles supported by OER acknowledge a NOAA grant number instead of the granting office, searching for OER grant numbers allows for the identification of additional articles. In this round, each NOAA grant number assigned to OER was searched for using Google. Because Google indexes the majority of publishers’ full text records, and because of the uniqueness of NOAA grant numbers, it could be used as a federated search engine in this case.

### Article analysis

Articles identified using both identification methods were compiled into a single database and duplicate articles were removed. In order to facilitate analysis, each article was then uniquely identified in WoS. WoS was selected as the data source due to the breadth and quality of its metadata indexing, the ability to perform citation analyses using WoS data, the comparatively poor quality of metadata and citation data in Google Scholar (Aguillo 2011; Jacso 2010), and because Scopus is not available at NOAA. 54 articles identified as being supported by OER, but that were still in press or published in journals not covered by this edition of WoS had to be excluded from this analysis. This included 16 articles in the field of underwater archaeology.

A custom result set consisting solely of articles previously identified as having been supported by OER was then created in WoS. The result set was then analyzed using the analysis tools built into WoS to identify the number of articles produced per year, the institutions that produced them, and their WoS-defined subject categories. Article metadata and citation data was then downloaded for further analysis. A list of articles citing OER-supported articles was then obtained and analyzed to identify the subject categories and institutions citing OER-sponsored articles. All citation data gathered during this process were accurate as of 02 April 2012.

Article records were then loaded into the Science of Science (Sci2) Tool (Sci2 Team 2009) for further analysis. Sci2 was used to create a geographic map of the US institutions that published OER-supported articles. It was also used to create and visualize a bibliographic coupling network and a word co-occurrence network from these articles. Technical details concerning the creation and visualization of these three maps will be given as each map is discussed below.

## Results and discussion

A total of 409 OER-supported articles were identified in WoS. A set of basic bibliometric indicators calculated for these articles are summarized in Table 1.

**Table 1:** Summary bibliometric indicators calculated for OER-supported articles

Bibliometric indicator	Value
Number of publications	409
Number of citations	4,219
Average citations per paper	10.32
H-index	30

171 (42 %) of these articles were first identified by the author-identification method, whereas 238 (58 %) were first identified by the search-based method. The search-based method also identified the majority of the publications originally identified by the author-identification method, making it the more comprehensive of the two methods used in this analysis. It cannot be recommended for use to the exclusion of the author-identification method, however, since the search-based method is unable to identify articles that do not specifically acknowledge OER support.

### Publication and citing article analysis

**Fig. 1:**
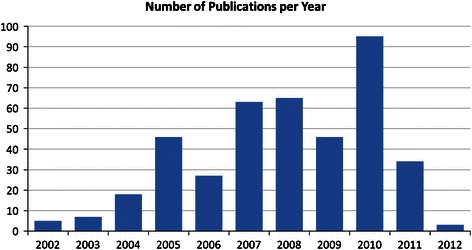
Number of OER-supported articles published per year (2002 to April 2012)

A publication trend analysis of these articles over time is presented in Fig. 1. As is evident, the production of OER-supported articles over time is highly variable, fluctuating, for example, from 46 articles in 2009 to 95 articles in 2010 and back to 34 articles in 2011. This fluctuation may have been caused by the publication in 2010 of three special issues of *Deep*-*Sea Research Part II* (volume 57; issues 1–2, 21–23, and 24–26) that were devoted in part or entirely to expeditions supported by OER.

Fluctuations in article production may also have been caused by fluctuations in OER’s grant budget, although establishing a connection between the two is problematic. Many of the articles identified as being supported by OER either could not be matched to a specific grant, or were supported by multiple OER grants over multiple years. Further complications arise from the fact that some articles resulting from a grant were published up to 10 years after the grant was awarded, although the majority of the publications that were successfully matched to a grant were published within 5 years of the award date.

**Fig. 2:**
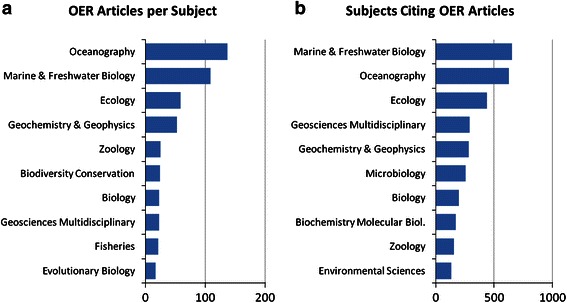
WoS subject categories of OER articles and articles citing OER articles

A comparison between the subject categories in which OER articles were published and from which OER articles received citations is presented in Fig. 2. These subject categories were assigned to articles by WoS based on the journal in which these articles were published. Note that articles assigned to multiple subject categories by WoS are counted multiple times in these figures, not fractionally. For clarity, only the top ten subject categories for each list are shown.

As is to be expected for a grant funding agency that funds exploration of the deep ocean, both OER articles and articles citing OER articles are dominated by publications in the categories of Oceanography and Marine and Freshwater Biology and in other related fields such as Ecology, Zoology, and Biology. Also strongly represented in both lists are the Geosciences, reflecting OER’s commitment to funding research on underwater geothermal processes.

Interestingly, the categories of Microbiology and Biochemistry and Molecular Biology are highly ranked in the citing articles list, but not highly ranked in the OER articles list. This suggests that some OER publications categorized by WoS in Oceanography and/or Marine and Freshwater Biology are actually microbiology articles published in more generalized journals. The high representation of microbiology in the citing articles list might also reflect the higher citation potential in this field.

**Fig. 3:**
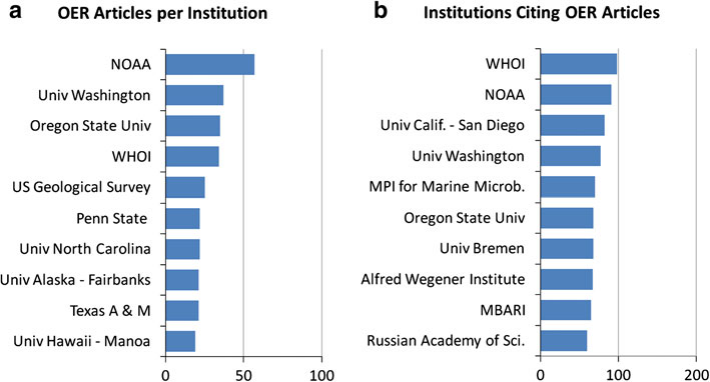
Comparison of institutions that publish and cite OER articles

A comparison of institutions that publish OER-supported articles versus institutions that cite OER articles is presented in Fig. 3. Publication and citation data for these publications were generated automatically by WoS and therefore should not be regarded as completely accurate. Also, publications produced by authors affiliated with multiple institutions are counted multiple times in this figure, not fractionally.

The publication list shows a high number of publications produced by NOAA authors, suggesting that although OER is technically an extramural funding agency, a large amount of OER funding supports internal NOAA science, or that NOAA authors are highly productive with the funding they receive, or both.

It is also interesting to note the high ranking of three German institutions—the Max Planck Institute for Marine Microbiology, the University of Bremen, and the Alfred Wegener Institute for Polar and Marine Research—in the citing articles list, but the relatively low ranking of these same institutions in the publishing articles list. This suggests that although authors from these institutions do not regularly publish with OER support, they do cite OER-supported work. This suggests that these institutions might be good candidates for participation in future jointly-funded expeditions with OER to explore areas of common interest.

### Geographic mapping

In order to better summarize the number of publications, number of citations, and geographic locations of institutions producing OER-supported research, a productivity and citation impact map of the US was created. To create the map, each of the 409 articles in this set was assigned to a single US zip code based on the article’s reprint address. This was done under the assumption that the reprint address indicates the author with the primary responsibility for the article in question. Articles missing a zip code in the reprint address were assigned to zip codes manually. Articles with a reprint address outside the United States were omitted.

**Fig. 4:**
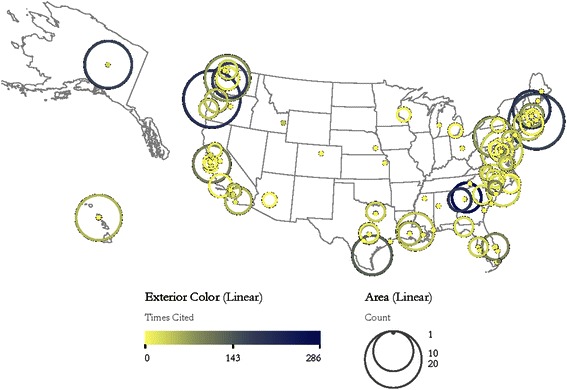
Location map of institutions producing OER-supported articles. *Circles* are centered over each zip code that produced at least one article. *Circle size* indicates the number of articles produced at that location; *circle color* indicates the total citation count of those articles (color figure online)

The articles were then aggregated based on common zip codes and the citation counts of those articles were summed. The generic geocoder built into the Sci2 software was then used to assign latitude and longitude values to each zip code and the resulting data was then mapped using Sci2’s geographic visualization tool. The resulting map is shown in Fig. 4.

343 of the 409 articles in this analysis listed a US address as their reprint address. Six articles listed zip codes that could not be geocoded, so a total of 337 (82 %) of the articles were successfully mapped. Due mostly to the fact that articles were assigned to a single address, rather than to all institutions listed, the institutional productivity levels shown on this map are slightly different than those identified by WoS and shown in Fig. 3.

The most productive zip codes are 97365 (Newport, OR) with 20 publications and 02543 (Woods Hole, MA) with 19 publications. The most highly cited zip codes are 30602 (Athens, GA) with 286 citations, 97365 (Newport, OR) with 197 citations, 03824 (Durham, NH) with 192 citations, and 30332 (Atlanta, GA) with 190 citations. The zip codes with the highest number of citations per paper, with of a minimum of five papers, are 30602 (Athens, GA) with 47.7, 98195 (Seattle, WA) with 22.125, 03824 (Durham, NH) with 21.3, and 34946 (Ft. Pierce FL) with 18.4.

Unsurprisingly, the zip codes shown on the map are distributed predominately in the coastal areas of the US. The map shows a high concentration of institutions on the East Coast, particularly between Massachusetts and South Carolina, and a relatively low concentration of institutions along the West and Gulf Coasts. However, institutions on the West and Gulf Coasts tend to have higher publication counts, suggesting that OER-supported publication in these areas tend to be concentrated in a few institutions, whereas publication by East Coast institutions tends to be more broadly distributed. The degree to which these publication patterns reflect the overall publication trends in oceanography and other marine sciences across the US is unknown.

### Bibliometric mapping

Bibliometric mapping is a complex network analysis (Albert and Barabasi 2002; Newman 2003) technique that attempts to analyze and visualize the structure inherent to a set of publications (Borner et al. 2003). Such maps are constructed by extracting data from a publication set and then creating networks based on co-occurrences, similarities, or other links in that data. Some examples of bibliometric mapping and network analysis include co-author networks (Newman 2001), paper citation networks (Boyack and Klavans 2010), journal citation networks (Franceschet 2012), and semantic networks (Mane and Borner 2004).

Bibliometric mapping has been shown to be useful in analyzing publications supported by grant-funding agencies (Boyack and Borner 2003; Noyons 2001; Rafols et al. 2010). In order to identify the major research areas represented in OER-supported research, a word co-occurrence and a bibliographic coupling network were created from the present set of OER publications. Both of these networks were generated, analyzed, and visualized using the algorithms included in the Sci2 Tool.

**Fig. 5:**
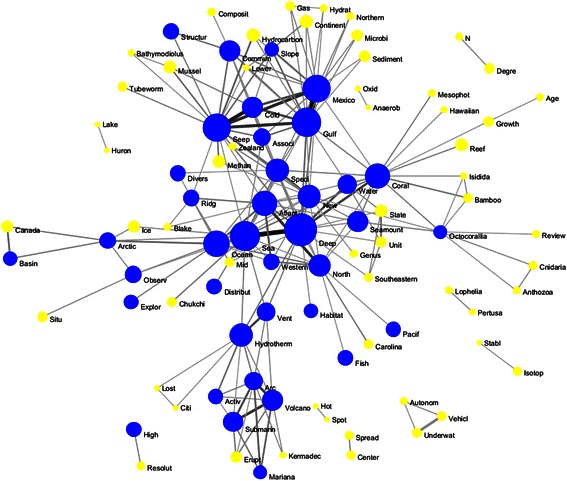
Title word co-occurrence network. Nodes are sized relative to the number of times the word occurs (range 5–72). Nodes representing words that occur 15 or more times are colored *blue*. Edges are sized and colored relative to the number of papers in which the two words co-occur (range 5–51) (color figure online)

The word co-occurrence network was created from words co-occurring in the titles of OER-supported articles. Words were stemmed and stop words were removed prior to creating the network. To increase the clarity of the final network map, the network was pruned by removing all edges with weight <5 and deleting all isolate nodes. The final network consists of 95 nodes, 204 edges, and 10 connected components. A map of this network is shown in Fig. 5.

The central terms in this network are “deep” and “sea,” with the two words co-occurring in 72 article titles. Arranged around these central terms are four clusters of related terms. Clockwise from the top of the map, these are Gulf of Mexico cold seeps, corals, underwater geophysics, and the arctic. Each cluster is anchored by one or more central terms and surrounded by additional terms identifying the subtopics within each research area. Within the corals cluster, for example, there seems to be a group of articles about Octocorallia, a subset of the class Anthozoa. Similarly, within the underwater geophysics cluster seems to be a subset of articles concerning the Lost City hydrothermal field.

To confirm these research areas, and to attempt to more clearly delineate the subtopics within each direction, a bibliographic coupling network was created from this publication set. Bibliographic coupling (Kessler 1963) was chosen for this analysis because of its accuracy in representing the structure of sets of scientific papers (Boyack and Klavans 2010), and because it can successfully map both cited and uncited articles, which co-citation mapping (Small 1973) cannot. This allows for the mapping of even the most recent, and therefore still uncited, articles supported by OER.

**Fig. 6:**
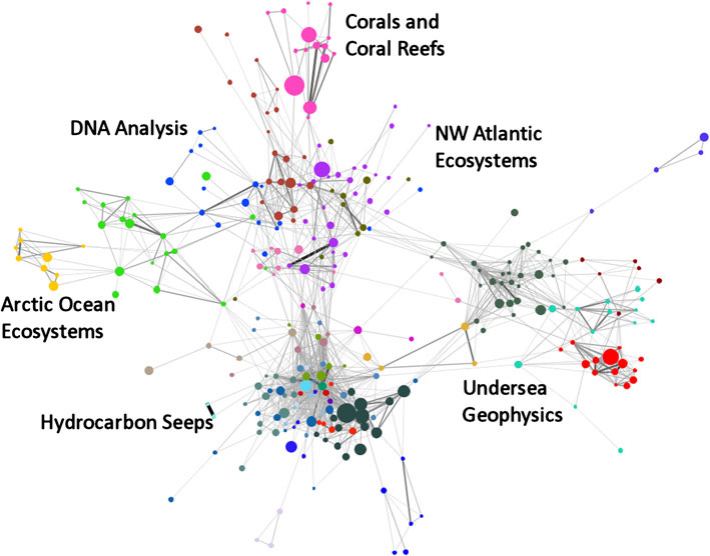
Bibliographic coupling network. Nodes are sized relative to the paper’s citation count (range 0–162) and* colored* based on the communities identified by the community detection algorithm. Edges are sized and colored relative to the number of shared references (range 3–41) (color figure online)

In order to increase the clarity of the final map, edges with weight <2 were removed and isolate nodes deleted. The largest connected component of the resulting network was then extracted. This component contains 300 (73 %) of the 409 OER-supported articles in the original publication set. This component was then analyzed using the community detection algorithm developed by Blondel et al. (2008) to identify network clusters. A map of the final network is shown in Fig. 6.

The final network displays clustering both at the network level and at the local level. Labels for network-level clusters were added to the map based on manual inspection of the articles in each cluster. Four of these network-level clusters, “Corals and Coral Reefs”, “Undersea Geophysics”, “Hydrocarbon Seeps”, and “Arctic Ocean Ecosystems” correspond to clusters identified by the word co-occurrence network, but two additional clusters, “DNA Analysis” and “NW Atlantic Ecosystems” were also identified. Together, these six clusters seem to identify the major research areas of OER-sponsored publications.

At the local level, the map shows evidence of clustering within these major research areas. Within the “Arctic Ocean Ecosystems” cluster are two distinct sub-networks concerning ecosystem processes (colored green on the map) and biogeochemistry (yellow) in the Arctic. Within the “Corals and Coral Reefs” cluster are sub-networks concerning genetic analysis of corals (brick red) and coral reef ecosystems (pink). Within the “Undersea Geophysics” cluster, there are four sub-networks: geophysical processes at seamounts and undersea volcanoes (dark green), geophysical monitoring and mapping of underwater ridges and spreading centers (teal), acoustic monitoring of undersea seismic activity (maroon), and explorations of the Lost City hydrothermal field (red). The majority of the publications in this cluster focus on undersea volcanoes and spreading centers in the Pacific Ocean explored during OER’s “Submarine Ring of Fire” series of expeditions.

The map also shows the connectivity between these research areas and sub-networks. At the network level, there seems to be a high degree of connectivity, or reference overlap, between the Hydrocarbon Seeps, NW Atlantic Ecosystems, DNA Analysis, and Corals and Coral Reefs clusters, but a rather low degree of connectivity between these clusters and the Arctic Ocean Ecosystems and Undersea Geophysics clusters. This low level of connectivity indicates possible knowledge gaps in these areas, at least in OER-supported publications. This suggests that OER might encourage research that connects these clusters, such as comparing the chemosynthetic communities in the Gulf of Mexico with those found at the Lost City Hydrothermal Field, exploring the effects of geothermal activity on deepwater ecosystems, or similar topics.

At the local level, there seems to be a low level of connectivity between sub-networks in the “Arctic Ocean Ecosystems” cluster. This suggests that OER might encourage research on ecosystem-wide processes in the Arctic, such as the effects of nutrient loading or primary production on Arctic Ocean food webs. Similarly, there seems to be little connectivity between articles on seamount morphology and those on seamount ecosystems, suggesting that additional research on the effects of seamount morphology on ecosystem function may be needed.

Finally, the map also depicts the concentration and raw citation impact of OER-supported publications in each of these research areas and sub-networks. The high concentration of articles and the large number of highly cited articles in the “Hydrocarbon Seeps” cluster suggest that OER support has resulted in a large body of relatively highly cited literature on this topic. By contrast, there are fewer articles in the coral reef ecosystems sub-network than in the “Hydrocarbon Seeps” cluster, but the percentage of highly-cited articles is higher. This suggests that although OER has not received a high degree of productivity on the topic of coral reef ecosystems, the articles that have been produced tend to be highly cited.

### Percentile analysis

Finally, in order to evaluate the citation counts of OER-supported articles, a percentile rank analysis was performed for articles in the WoS subject categories of Oceanography and Marine and Freshwater Biology, the two subject categories in which OER-supported articles were most often published. Percentile ranks were selected for this analysis based on the growing consensus that they are more stable and consistent than most of the bibliometric indicators currently available (e.g. Bornmann et al. 2012; Centre for Science and Technology Studies 2011; Leydesdorff et al. 2011). Note that only articles published in one or both of these categories were selected for this analysis and that articles published in both categories were counted as a full article in both categories, rather than fractionally.

Percentile thresholds and article percentile ranks were generated manually. First, all articles published in one of these categories and published during a single year were identified in WoS and then ranked in descending order by citation count. Then, the citation counts necessary for an article to be ranked in one of four percentile rank classes—99th, 90th, 50th, and <50th—were identified. This process was repeated for the years 2002–2010 in both subject categories. Due to the presence of multiple articles with the same citation count, particularly at the 50th percentile rank, these percentile thresholds were not always clearly delineated. Articles published in multidisciplinary journals (*Nature*, *Science*, *PNAS*, etc.) were omitted from this analysis because they are assigned to an aggregate subject category—Multidisciplinary Sciences—that could not be parsed into subject-specific categories. Next, OER-supported articles in Oceanography and Marine and Freshwater Biology were assigned percentile ranks by comparing their citation counts to the thresholds identified for their subject category and year of publication. Finally, the total number of articles in each of these percentile rank classes for each subject category was calculated.

**Fig. 7:**
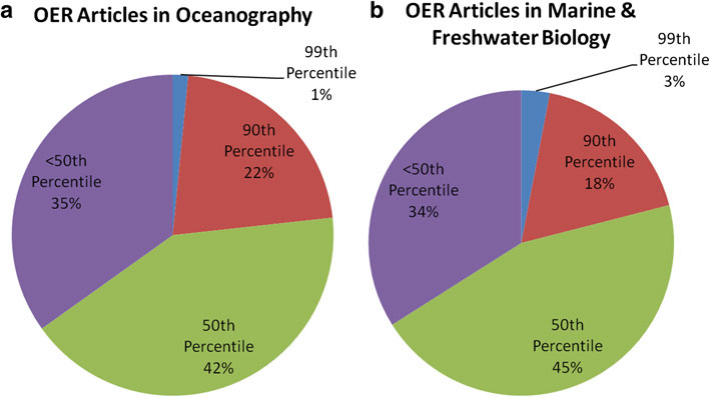
Percentile ranks of OER articles in Oceanography and Marine and Freshwater Biology

The results of this analysis are summarized in Fig. 7. Approximately 23 % of OER-supported articles in Oceanography and 21 % of OER-supported articles in Marine and Freshwater Biology were ranked in or above the 90th percentile for citation counts. These percentages are more than twice as high as would be expected from an average distribution. In addition, only 35 % of these articles in Oceanography and 34 % of these articles in Marine and Freshwater Biology were ranked in the <50th percentile rank class. These findings suggest that OER-supported articles in these two subject categories tend to be more highly cited than the average articles in these categories.

## Conclusions

This article analyzed the scientific journal articles supported by NOAA’s Office of Ocean Exploration and Research from 2002 to April 2012. 409 articles were analyzed, the majority of which (58 %) were identified through targeted searching, rather than through author identification. Article publication over time was found to be highly variable. Institutional analysis and geographic mapping found that OER funds both intramural and extramural research projects, that US institutions receiving OER support are located predominately in coastal areas, and that OER funding of institutions on the West Coast of the US is concentrated in fewer institutions than its funding of institutions on the East Coast.

Bibliometric mapping identified six major research areas of OER-supported publications: “Corals and Coral Reefs”, “NW Atlantic Ecosystems”, “Undersea Geophysics”, “Hydrocarbon Seeps”, “Arctic Ocean Ecosystems”, and “DNA Analysis”. A high concentration of OER-supported articles was found in the “Hydrocarbon Seeps” research areas, and highly cited articles were found to be common in the “Hydrocarbon Seeps” and “Corals and Coral Reefs” research areas. Percentile analysis found that a higher than expected percentage (over 20 %) of OER articles in the subjects of Oceanography and Marine and Freshwater Biology were ranked in the 90th percentile for their subjects and years of publication. The analysis also found that a lower than expected (around 35 %) percentage of these articles were ranked in the <50th percentile.

These conclusions are constrained by a number of limiting factors. Due to the limitations of the version of WoS used for this analysis, non-journal publications supported by OER and citations received by OER publications from non-journal publications could not be included in this analysis. The use of WoS also meant that most of the OER-supported publications in the social sciences, particularly in underwater archaeology, were also omitted from this analysis. It is also likely that the articles analyzed here are not a comprehensive list of OER-supported publications due to the lack of acknowledgement of OER support in some articles. Finally, the relatively short citation window (approximately 10 years) means that articles in disciplines like Oceanography, Marine and Freshwater Biology, and Multidisciplinary Geosciences, which have cited half-lives of 8–9 years, may not yet have had sufficient time to accumulate citations.

The analysis presented in this paper represents the first step in analyzing the scientific and technological contributions of OER. Future research could analyze the degree to which the publication output analyzed in this paper compares to the stated research priorities and funding decisions of OER. In addition, since OER’s mission includes the development of undersea research technology, an analysis of the patents developed with OER support would add a dimension of OER impact largely missing from the present analysis. Finally, an analysis of the publications and patents sponsored over the 30-year history of NURP would contribute to a more comprehensive understanding of NOAA’s contributions to oceanographic research.
